# Structural Modulation of TpPa‑1 Covalent Organic Framework in Flow Synthesis Guided by Green Chemistry Principles

**DOI:** 10.1021/acsengineeringau.5c00114

**Published:** 2026-04-17

**Authors:** Yizhuo Xu, Nikita Rog, Catherine Mollart, Ganna Gryn’ova, Abbie Trewin, Cher Hon Lau

**Affiliations:** † School of Engineering, 3124 The University of Edinburgh, Edinburgh EH9 3FB, The United Kingdom of Great Britain and Northern Ireland; ‡ School of Chemistry, 1724 University of Birmingham, Birmingham B15 2TT, The United Kingdom of Great Britain and Northern Ireland; § Department of Chemistry, 4396 Lancaster University, Lancaster LA1 4YB, The United Kingdom of Great Britain and Northern Ireland

**Keywords:** Covalent organic frameworks (COFs), Green chemistry, SMD simulation, Continuous-flow synthesis, Free energy of solvation, Hansen solubility parameters

## Abstract

Here we present a solvent decision-making pathway for less hazardous and scalable synthesis of TpPa-1 covalent organic framework (COF) underpinned by environmental compatibility (CHEM21 framework), processability (Hansen solubility parameters), and thermodynamic suitability (free energy of solvation). Our analyses suggest that solvents with moderate solvation, such as 1,4-dioxane, propylene carbonate, and diacetin, hinder excessive reaction reversibility and promote crystallite growth. Balancing solvent greenness, compatibility, and thermodynamics, we find that diacetin is optimal for a continuous-flow synthesis. Compared with TpPa-1 COF synthesized in diacetin under batch conditions, the Brunauer–Emmett–Teller surface area of flow-derived TpPa-1 reached 418 m^2^ g^–1^, increasing CO_2_ uptake by 50% at 298 K. The flow process achieved a 30-fold increase in space-time yield (STY), with excellent reproducibility and an 89% reduction in specific energy consumption. This solvent-thermodynamics-guided approach establishes a physically transparent and sustainable framework for optimizing COF synthesis under continuous-flow conditions.

## Graphical abstract

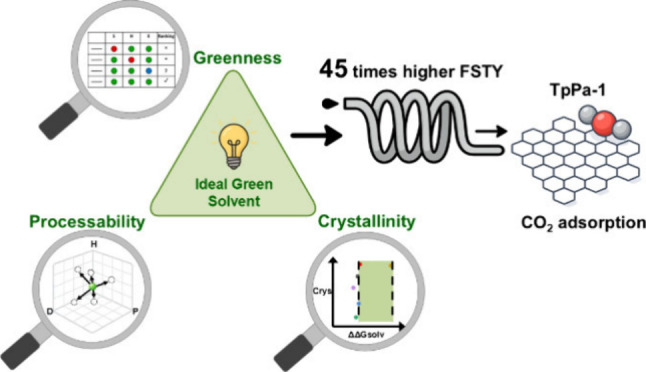

## 1 Introduction

Covalent organic frameworks (COFs) are crystalline porous polymers built from light elements through strong covalent bonds, combining high surface area, ordered pore channels, and structural tunability in one platform.^1–4^ First reported in 2005, COFs have been applied in gas storage, separation, catalysis, and sensing due to their high specific surface area and structural programmability.^5^ Further research in this field has established the principle that strongly bonded systems require controlled reversibility to facilitate self-repair during crystallization. However, in COF polymerization, there exists a trade-off among reversibility, crystallinity, and stability. Enhancing bond reversibility promotes self-repair and rearrangement, thereby assisting the growth of long-range-ordered crystals, while excessive reversibility compromises the chemical and thermal stability. In contrast, stronger linkages significantly improve robustness, which is also attributable to their resistance to self-repair, resulting in amorphous networks with lower crystallinity. This trade-off has been identified by multiple studies as a core challenge within the COF field.^6–8^

TpPa-1 is a typical β-ketoenamine-bonded COF, constructed through Schiff base condensation between 2,4,6-Trihydroxybenzene, 1,3,5-triformylphloroglucinol (Tp), and *p*-phenylenediamine (Pa-1) (Figure 1). Such β-ketoenamine linkages offer both the initial reversibility provided by imine exchange and the product stability provided by tautomeric locking.^9–11^ These frameworks display exceptional resistance to acid–base degradation, making TpPa-1 one of the most frequently studied COFs in this category.^10^

**Figure 1:**
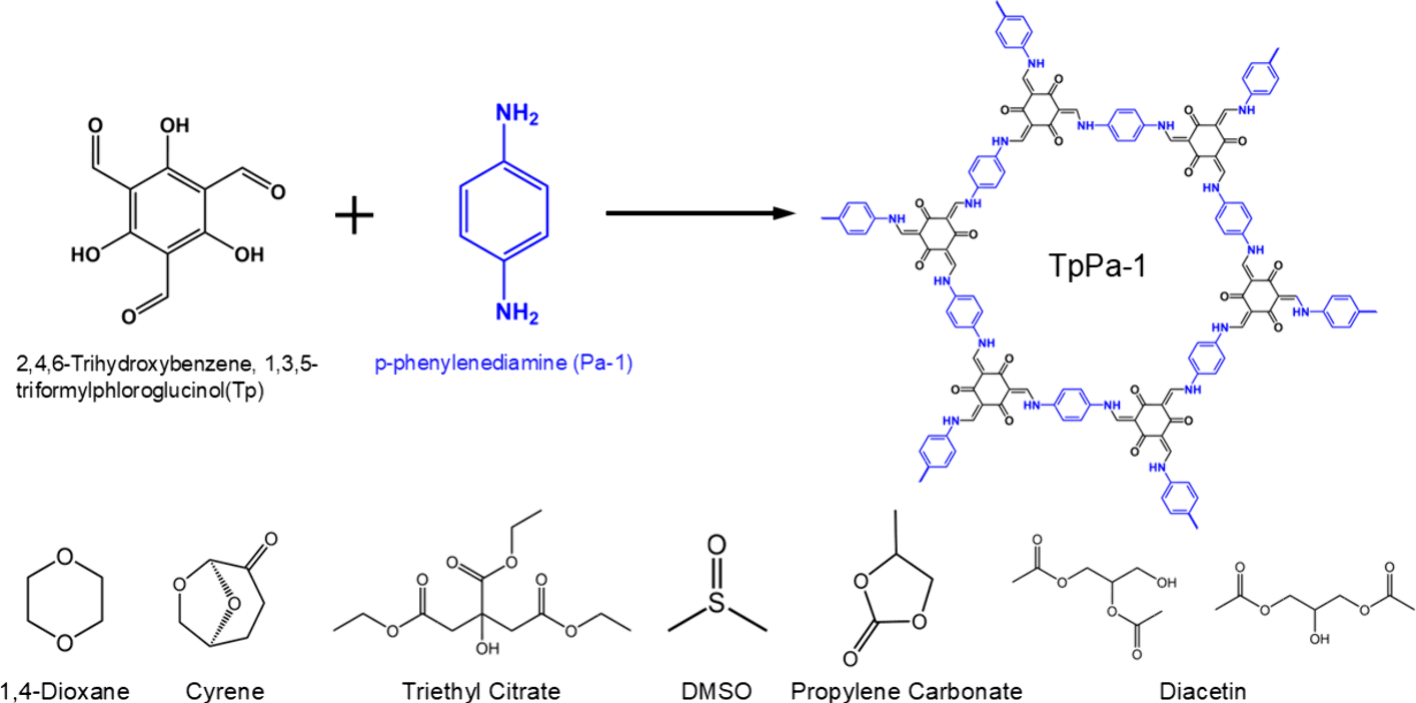
Top: synthesis of TpPa-1 COF from 2,4,6-Trihydroxybenzene, 1,3,5-triformylphloroglucinol (Tp), and *p*-phenylenediamine (Pa-1). Bottom: structure of the solvents used to synthesize TpPa-1. Diacetin is a 1:1 mixture of 1,2- and 1,3-glyceryl diacetate, and both structures are therefore shown in the figure.

Beyond the choice of linkage, the solvent has a strong influence on how COFs nucleate and grow. Solvent polarity and hydrogen-bonding interactions affect the reversibility of imine exchange and rearrangement, thereby influencing critical nucleation density and domain growth rates. Meanwhile, the viscosity and solubility parameters collaboratively determine transport behavior, nucleation uniformity, and early microstructure formation during synthesis. Together, these factors define the window for achieving optimal crystallinity.^12–14^ Moreover, solvents can energetically improve self-repair through the selective solvation of monomers or intermediates, thereby inducing interlayer slipping and stacking rearrangement. As a result, the crystallinity and interlayer order of the same COF can be drastically different when it is synthesized in different solvents following the same overall reaction.^15–18^

When solvent performance is assessed, environmental sustainability is an equally critical factor. The CHEM21 Solvent Selection Guide provides a systematic evaluation of solvents based on safety, health, and environmental (SHE) criteria, offering an effective foundation for identifying biobased, low-toxicity alternatives.^19^ In parallel, Hansen solubility parameters (HSPs) characterize solvent–solute affinity through a three-dimensional coordinate system of dispersion (δd), polarity (δp), and hydrogen bonding (δh). By calculating distances within Hansen space, monomer–solvent compatibility can be estimated, guiding solvent selection and mitigating clogging risks during scale-up, which is an especially relevant concern in flow chemistry.^20,21^

Following the initial sustainability and compatibility screening, the free energy of solvation (*ΔΔG_solv_*) provides a crucial parameter for transitioning from empirical solvent selection to thermodynamic quantification. *ΔΔGsolv* represents the free energy change when a solute transitions from the gas phase to the solvent phase, which can be computed across a wide range of solvents using the implicit Solvation Model Density (SMD) model. For dynamic crystallization of COFs, a more negative free energy of solvation typically indicates stronger stabilization of reactants or intermediates via interactions with the solvent and a higher desolvation cost, establishing an equilibrium between self-repair and crystalline phase growth. Excessively strong solvation will induce excessive reversibility, hindering adequate domain growth. On the contrary, insufficient (weak) solvation leads to inadequate reversibility, preventing complete defect elimination within the lattice.^23–25^ Therefore, solvent selection based on the *ΔΔG_solv_* ranking, combined with structural characterization interpretation, elevates solvent choice beyond apparent solubility to thermodynamic suitability and crystalline phase order.

Traditionally, COFs have been synthesized through batch solvothermal methods. However, the use of green solvents in COF synthesis is frequently accompanied by a decrease in porosity and CO_2_ adsorption, reflecting an intrinsic trade-off between environmental sustainability and performance.^26,27^ Recently, continuous-flow synthesis has gained increasing attention as a sustainable alternative, offering enhanced control over reaction parameters, improved reproducibility, and facile scalability.^28,29^ Studies on flow synthesis of metal organic frameworks (MOFs), hyper-cross-linked polymers (HCPs), and COFs indicate that appropriately designed continuous-flow platforms can mitigate or even overcome this trade-off, enabling the use of greener solvents while retaining competitive performance.^30–32^ Integrating green solvent systems into flow-based COF synthesis represents a promising step toward environmentally benign and industrially viable production. In this work, we demonstrate a continuous-flow approach for the synthesis of COFs using less hazardous solvents, providing an efficient, sustainable, and reproducible pathway to high-quality COF materials.

This paper employs the SMD continuous solvent model to estimate the *ΔΔG*_*solv*_ for the Tp monomer, which dominates the dissolution process during synthesis.^33^ Using this as the core variable, we constructed an analytical framework linking HSP compatibility to crystallinity. Within this semiquantitative framework, we identify trends in crystallization quality across less hazardous solvents and pinpoint their optimal window. Supplemented by CHEM21 screening, we identified diacetin as the optimal solvent for flow synthesis of COFs, with propylene carbonate (PC) serving as a performance upper limit for comparison. Through iterative optimization, we achieved optimal experimental configurations, yielding flow-synthesized powders comparable in performance to solvothermal synthesized powders.

## 2 Experimental Section

### 2.1 SMD Calculations

After optimizing the geometry of the Tp monomer in the gas phase, the free energy of its solvation in each of the studied solvents at 25 °C was obtained using the SMD implicit solvation model and the M06–2X/cc-pVTZ model chemistry with Gaussian 16.^22,34–36^ Full details of the parameters used to define the SMD model for each solvent are given in Table S1.

### 2.2 Solvothermal Synthesis

The TpPa-1 COF powder was synthesized as follows. The reaction was implemented with Tp (63 mg, 0.3 mmol) and Pa-1 (48 mg, 0.45 mmol) using different solvents (3 mL) in the presence of 3 M acetic acid (0.5 mL). All components were added to a thick-walled pressure tube. After undergoing three freeze–thaw cycles, the tube was sealed, and the reaction proceeded at 120 °C in an oil bath for 72 h. The product was washed repeatedly with water and ethanol and then dried under vacuum at 80 °C to yield a red TpPa-1 powder.

### 2.3 Flow Synthesis

TpPa-1 was also synthesized using a continuous-flow setup equipped with a perfluoroalkoxy alkane (PFA) tubing reactor (inner diameter 1 mm, length 7.6 m, and total volume 6 mL) placed in a reactor coil heater and connected to a syringe pump for reagent delivery. A back pressure valve was installed to regulate the pressure inside the reactor (Figure 3A).

In a typical experiment, two separate precursor solutions were prepared: Solution A contained 126 mg of Tp dissolved in 13 mL of diacetin, and Solution B contained 96 mg of Pa dissolved in 11 mL of diacetin and 2 mL of 3 M acetic acid. The two solutions were delivered into a T-mixer at controlled flow rates of 0.1 mL min^–1^ each, ensuring a combined residence time of 30 min in the reactor coil. The reactor coil was maintained at 150 °C and 5 barg.

The outflow from the reactor was collected continuously and cooled to room temperature. The resulting suspension was separated by centrifugation, and the solid product was washed with ethanol to remove the unreacted precursors and residual impurities. The obtained powder was dried under a vacuum at 80 °C overnight to yield a red TpPa-1 powder.

## 3 Results and Discussion

### 3.1 Greenness Assessment and Process Safety

In addition to the conventional solvent 1,4-dioxane (dioxane), we selected five less hazardous solvents. The selected solvents were classified according to the CHEM21 evaluation as shown in Table 1. Apart from their more favorable health and environmental properties compared with dioxane, these less hazardous solvents were chosen to be compatible with the high-temperature continuous-flow synthesis of TpPa-1. Their relatively high boiling points and low vapor pressures allow stable operation under high pressure by avoiding the formation of vapor plugs in the reactor. Moreover, under the mildly acidic reaction conditions, they are sufficiently inert to avoid participating in side reactions with the aldehyde or amine monomers. Diacetin is synthesized from glycerol containing three hydroxyl groups. Consequently, the acetylation of only two of these groups yields two isomers.

**Table 1:**
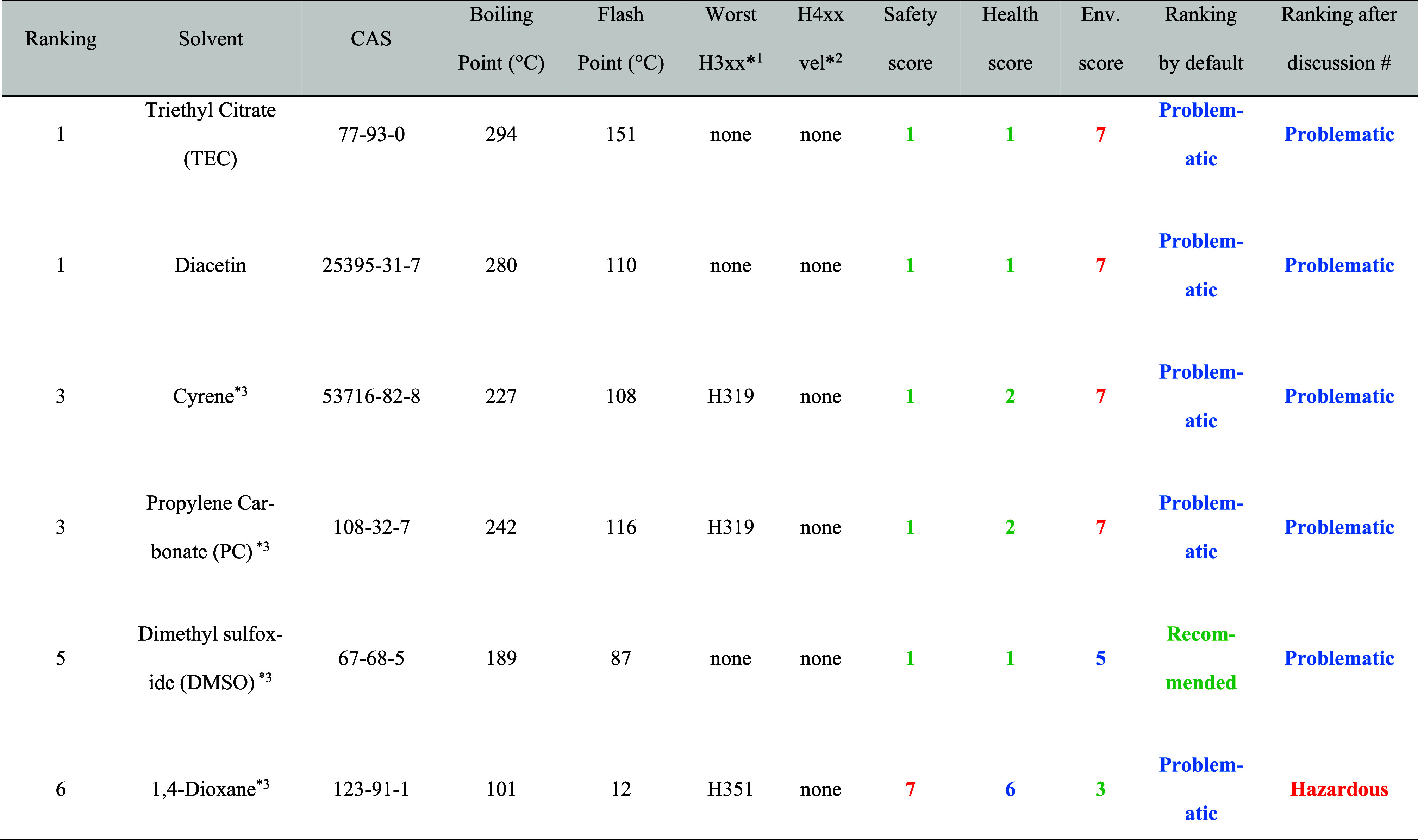
CHEM21 Green Metrics of Solvents Used in This Work^a^

a *1 represents the most severe GHS health hazard statement for solvents (H3xx code). *2 represents the most severe GHS environmental hazard (H4xx). *3 SHE values and ranking were compiled from literature values.^19^ Different colors represent the following: green - recommended, blue - problematic, red - hazardous.

Among the six solvents used in this study, diacetin and triethyl citrate (TEC) exhibit excellent health and safety properties. High flash points and low volatility make them suitable for closed system heating and continuous-flow pressurized applications. Since diacetin and TEC are not listed in the published CHEM21 tables, we rebuilt their Safety (S)/Health (H)/Environment (E) scores from primary data. The S+H+E index for both solvents is 1, 1, and 7. Where a solvent’s SHE scores fall into different bands, we follow the CHEM21 precautionary rule that the worst band determines the overall category.^37^ Thus, these two biobased solvents are classfied as problematic under the CHEM21 criteria, mainly due to their high boiling points which could cause higher energy demand for solvent removal. In the context of flow synthesis, however, their high boiling points and low volatility are advantageous by allowing the monomers to remain in the same liquid phase, reducing vapor formation, and avoiding blockages. Additionally, because their long-term toxicological and regulatory data are relatively limited, a conservative rating is preferred.

PC and cyrene are considered joint third. Both solvents are dipolar aprotic liquids with relatively high boiling points and low vapor pressures and have been considered as greener alternatives to classical polar aprotic solvents such as DMF or NMP, providing adequate solubility for aromatic aldehydes and amines.^38,39^ Dimethyl sulfoxide (DMSO), though considered a safer laboratory solvent in most cases, is classified as higher risk due to its permeation properties and ability to enhance skin absorption in practical applications.^40^ 1,4-Dioxane is explicitly marked as not recommended due to its health hazards.^19,37^ As such, we did not perform any continuous-flow experiments with 1,4-dioxane.

### 3.2 Solvent Fingerprint and Crystallization Tendency

Given that Tp dominates the dissolution process during synthesis, we focused on the distance between each solvent and the Tp monomer within the Hansen Solubility Parameters in Practice (HSPiP) 3D sphere (Figure 2A).^33^ DMSO has the smallest Tp-distance, followed by diacetin, cyrene, TEC, and PC, while dioxane has a much larger Tp-distance as a conventional solvent (Table 2). From the perspective of flow synthesis feasibility, a smaller HSP value difference indicates better dissolution and reduced susceptibility to clog the reactor. Consequently, diacetin possesses inherent advantages in flow synthesis.

**Figure 2:**
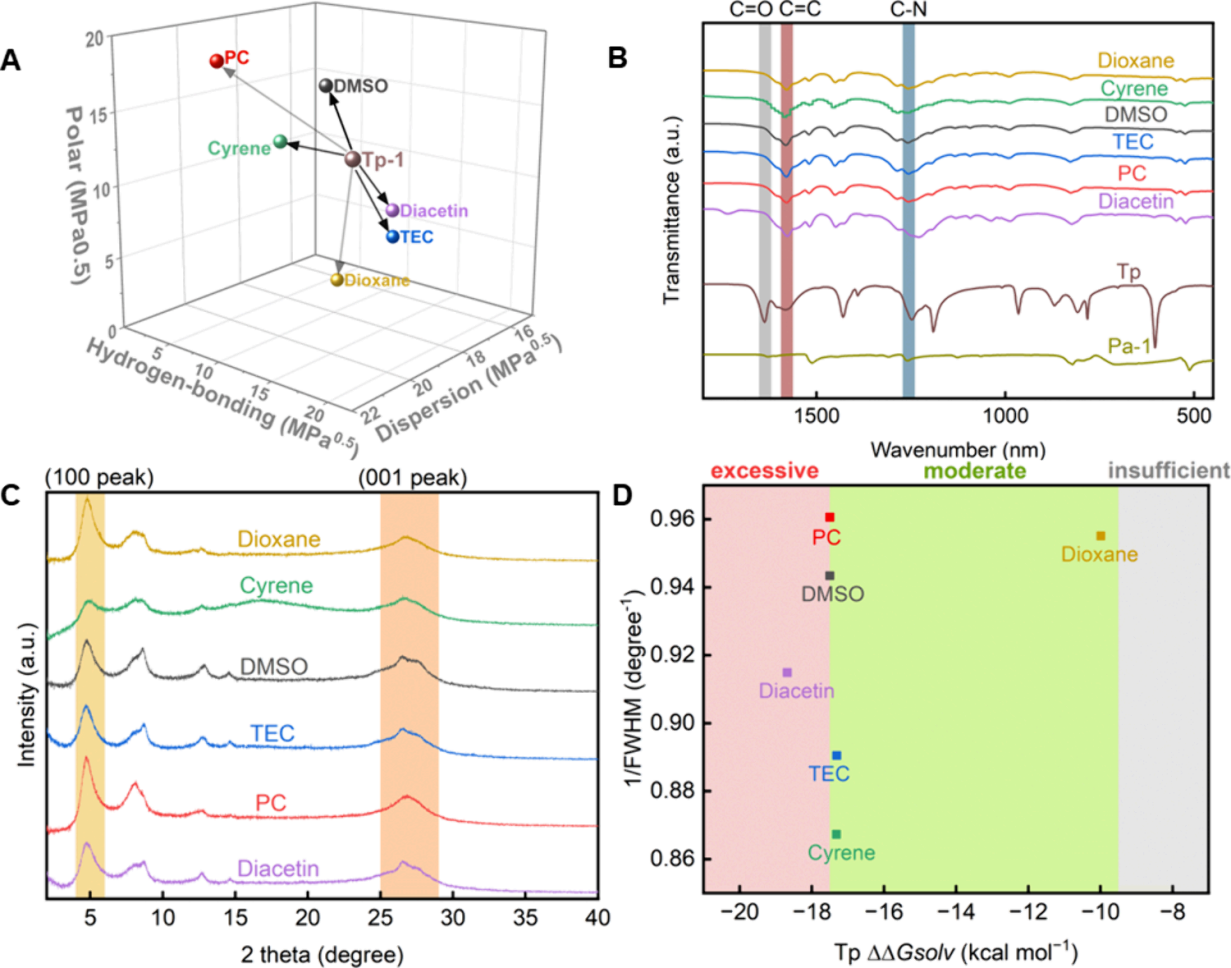
Solvent Fingerprint and powder properties. (A) Tp monomer (green sphere) and solvents (blue spheres) in a HSPiP 3D sphere. (B) FTIR spectra for products synthesized by the solvothermal method and monomers. (C) PXRD patterns for products synthesized by the solvothermal method. (D) Scatter plot of 1/fwhm versus Tp *ΔΔG*_*solv*_ for the six products and reversibility regions. For B, C, and D, the solvents used to synthesize TpPa-1 are labeled.

**Table 2:** Sample Channel Characterization

Solvent	HSPiP Tp distance (MPa^0.5^)	1/fwhm (100) (degree^–1^)	Tp *ΔΔG*_*solv*_ (kcal mol^–1^)
Dioxane	18.30	0.955	–9.99
Cyrene	13.76	0.867	–17.32
Triethyl Citrate	15.59	0.890	–17.31
DMSO	11.36	0.943	–17.50
Propylene Carbonate	16.36	0.961	–17.52
Diacetin	12.30	0.915	–18.68

The *ΔΔG*_*solv*_ of Tp shown in Table 2 reflects the degree of stabilization of the Tp monomer by the solvent. A more negative Tp *ΔΔG*_*solv*_ indicates that Tp is more strongly bound by that solvent, and the energy cost of desolvation before reaction will be higher.^41^ The HSP model estimates solubility by considering three parameters of both solvent and solute: dispersion force, polarity, and hydrogen bonding, which aligns with the objective of *ΔΔG_solv_*: overall, the two parameters exhibit a consistent trend.

As shown by the Fourier transform infrared (FTIR) spectra in Figure 2B, the aldehyde C=O (∼1665–1690 cm^–1^) peak disappears in all six samples, while the new C=N/C=C (∼1620–1640 cm^–1^) and C–N (∼1240–1280 cm^–1^) peaks characteristic of β-ketoenamine linkage appear. The FTIR spectra confirm that the TpPa-1 reaction was complete in each solvent synthesis, yielding the expected structures. The powder X-ray diffraction (PXRD) patterns in Figure 2C reveal that all six samples exhibit sharp (100) main reflection peaks near 5°, along with broad (001) interlayer peaks between 25–30°. The PXRD peak positions and diffraction profile are consistent with literature-reported TpPa-1, confirming preservation of the framework structure.^10,42–44^

The appropriate reversibility of synthesizing covalent organic frameworks has been emphasized by many studies, which can be intuitively reflected by the reaction equilibrium constant *K*. The relationship between *K* and reaction free energy *ΔG* ^0^ is as follows

$$\Delta G^{{}^{\circ}}=-RT\;\ln\;K$$ 1

where *R* is the universal gas constant, and *T* is the absolute temperature. The standard reaction free energy in solution can be decomposed into the sum of the gas-phase intrinsic term and the solvation term:

$$\Delta G_{rxn,so\ln}^{0}=\Delta G_{rxn,gas}^{0}+\Delta G_{rxn,solv}$$ 2

Among all of the reactants, Tp is the least soluble. We therefore regard its solvation free energy as an upper bound to the energy cost of dissolution in this system. In this sense, the *ΔΔG*_*solv*_ of Tp provides a convenient descriptor of the solvation contribution to the overall reaction free energy ΔG^0^. Combining this with eq 1, we expect equilibrium constant *K* to increase as the *ΔΔG*_*solv*_ of Tp becomes more negative. According to the Scherrer equation

$$L_{c}=\frac{K\lambda }{\beta \;\cos\;\theta }$$ 3

$$\frac{1}{fwhm}\propto L_{c}$$ 4

The crystallite domain size is inversely proportional to the full width at half-maximum (fwhm) of the (100) diffraction peak. We therefore use 1/fwhm as a qualitative measure of structural ordering in the following analysis.^45,46^ In 2D COFs, peak width reflects not only grain size but also stacking disorder and microstructural variations. Therefore, the 1/fwhm value serves only for relative comparisons between samples measured under identical conditions.^47^ As shown in Table 2, the 1/fwhm values for all products were plotted against their relative Tp *ΔΔG*_*solv*_ (Figure 2D). The calculation method for fwhm is detailed in the SI. This revealed that reversibility increased as Tp *ΔΔG*_*solv*_ became more negative with 1/fwhm exhibiting an overall decreasing trend. Relatively, PC and dioxane fall within the moderately reversible region, exhibiting higher 1/fwhm values. By contrast, cyrene, TEC, and diacetin enter the excessively reversible zone, resulting in lower 1/fwhm values.

However, we believe that the incorporation of a less hazardous solvent as a replacement for the more hazardous conventional dioxane can be further improved for a greener synthesis of TpPa-1 with particular emphasis on the Green Chemistry Principles of safer solvents and auxiliaries (Principle 5), energy efficiency (Principle 6), and inherently safer chemistry for accident prevention (Principle 12). A solvothermal batch reaction has a relatively high energy demand and low space-time yield. A continuous-flow setup (Figure 3A) can overcome these disadvantages thanks to its thermal efficiency and scalability. Thus, we aim to make TpPa-1 synthesis as green as possible by using an energy-efficient continuous-flow process and by choosing less hazardous solvent systems that reflect these principles.

**Figure 3:**
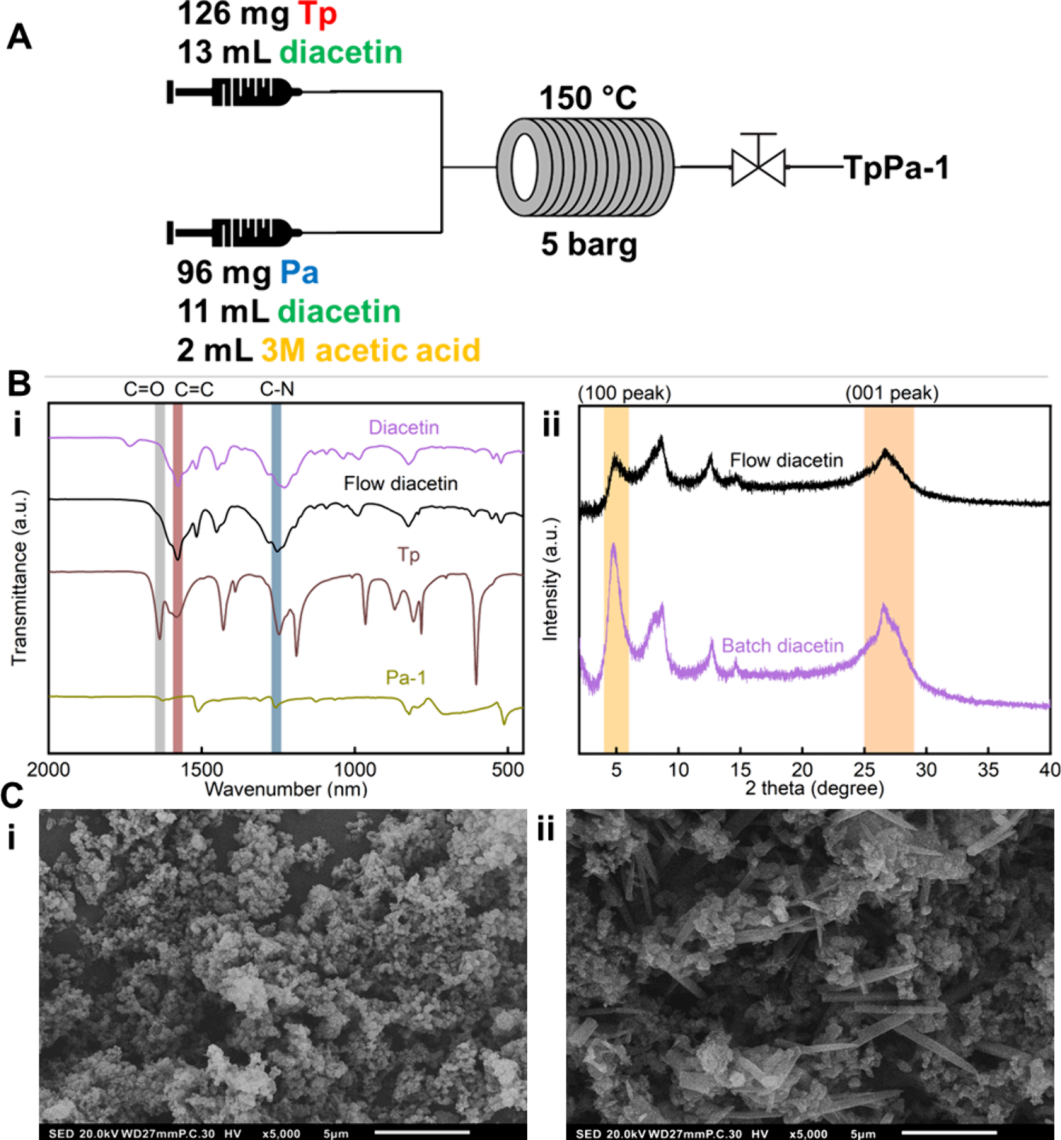
(A) Flow synthesis procedure; (B) Flow synthesis powder properties. (i) FTIR results, (ii) XRD results; (C) SEM results of diacetin powders synthesized by (i) Flow chemistry, (ii) Thermochemical method.

Based on this analysis, the solvothermal results were used to select and validate solvent candidates for flow synthesis through a semiquantitative trend. TpPa-1 synthesized using PC shows the best crystallinity among the tested solvents, while the same material synthesized in diacetin combines reasonably high crystallinity with the most favorable environmental profile. At the same time, solvothermal synthesis in these less toxic solvents led to TpPa-1 samples with lower porosity and reduced CO_2_ adsorption capacity than the dioxane benchmark, indicating that improved sustainability is achieved at the expense of materials performance. These two solvents were therefore chosen as the best candidates for flow chemistry, in order to test whether continuous-flow synthesis can compensate for this loss in porosity and CO_2_ uptake, previously demonstrated by other works.^48,49^ In the next section, we examine how solvent choice and varying reaction conditions together influence both crystallinity and overall practicality of flow setup.

### 3.3 Flow Synthesis Analysis

The continuous-flow synthesis of TpPa-1 COF was systematically optimized by varying key reaction parameters, including the residence time, monomer concentration, catalyst loading, temperature, and system pressure (Table S2). The optimization aimed to maximize the Brunauer–Emmett–Teller (BET) surface area (SA) while maintaining operational stability of the flow reactor.

A key practical consideration in this work was the choice of the solvent. Diacetin was selected as the reaction medium instead of PC, as it provided improved solubility for the Tp monomer and ensured a stable flow operation. In PC, Tp exhibited limited solubility at ambient temperature, leading to precipitation and clogging of the reactor tubing during extended operation. In contrast, diacetin enabled homogeneous mixing of both monomers throughout the reaction course, resulting in continuous, uninterrupted synthesis, and reproducible material properties.

Monomer concentration played a decisive role in the reactor performance and product quality. Higher concentrations of Tp and Pa-1 resulted in poor dissolution of the monomers, leading to clogging or less crystalline products. An optimal balance was achieved at a 4-fold monomer dilution ratio to the batch conditions, providing a stable suspension and uniform product formation.

Catalyst loading was another important parameter with a significant effect on the product surface area. Once the monomer dilution was set to 4×, varying amounts of acetic acid were tested (1×, 2×, 4×, and 8× relative to the batch analogue), with 2× dilution yielding the highest quality TpPa-1. Similarly, temperature screening from 110–150 °C revealed that heating at 150 °C resulted in a product with higher BET SA. It should be noted that the choice of maximum limit of 150 °C was due to the maximum operating temperature of the reactor’s PFA tubing.

The addition of a back-pressure regulator greatly improved the quality of the TpPa-1 product. At elevated pressure, water and acetic acid have higher boiling points than the reactor temperature, resulting in stable flow inside the reactor coil. Among the tested conditions, shorter residence times (<10 min) led to incomplete conversion, as evidenced by lower BET surface areas. Increasing the residence time to 30 min significantly improved framework formation, suggesting that sufficient condensation time is critical for nucleation and growth of TpPa-1. Under the optimized conditions, the reactor operated continuously for 4 h without observable clogging.

The structure and crystallinity of flow-synthesized TpPa-1 COF were confirmed by FTIR and PXRD analyses (Figure 3B). The FTIR spectrum (Figure 3Bi) exhibits characteristic bands at approximately 1620–1640 cm^–1^ and 1240–1280 cm^–1^ corresponding to the C=N/C=C and C–N β-ketoenamine vibrations, respectively. The absence of significant peaks at 1665–1690 cm^–1^ indicates near-complete consumption of aldehyde and amine functional groups, respectively. These spectral features closely match those of the batch-synthesized counterpart, demonstrating that the continuous-flow process preserves the chemical integrity of the COF framework. PXRD analysis (Figure 3Bii) revealed well-defined diffraction peaks at 5° and between 25–30°, corresponding to the (100) and (001) planes with peak positions being consistent with those observed in the batch sample, indicating that the flow-synthesized COF retained the same crystalline structure. X-ray photoelectron spectroscopy (XPS) was employed to evaluate the elemental composition of TpPa-1 synthesized in diacetin via batch and flow configurations. For the batch-made diacetin TpPa-1, the atomic concentrations were determined to be 75.28% C, 14.13% O, and 10.60% N. Similarly, the flow-synthesized counterpart exhibited an elemental composition of 74.68% C, 15.63% O, and 9.69% N. Both samples align closely with the theoretical C/N/O stoichiometric ratio of 15:2:3, confirming the compositional consistency of TpPa-1 across different synthesis routes.

In the SEM images (Figure 3C) of TpPa-1 synthesized in diacetin, the batch product (Figure 3Cii) exhibits noticeable spike-like features, whereas the flow TpPa-1 (Figure 3Ci) shows much smaller, mostly flaky particles. This morphological shift likely arises from differences in the nucleation and growth dynamics between the two synthetic routes. In batch synthesis, slower and more heterogeneous mixing in viscous diacetin can lead to anisotropic, diffusion-limited overgrowth, resulting in a spiky particle morphology. On the other hand, flow synthesis imposes rapid and uniform mixing, which promotes a high nucleation rate and suppresses prolonged directional growth, yielding many small nuclei that grow isotropically.^50^ Furthermore, shear and residence time effects in flow can kinetically limit the directional growth of crystals, reducing the characteristic anisotropic features that appear in batch conditions.^51^ Collectively, these results confirm that the optimized continuous-flow conditions afford high-quality TpPa-1 COF materials comparable to those produced via conventional batch synthesis while offering the additional advantages of scalability, improved reproducibility, and lower energy consumption.

### 3.4 BET Analysis and CO_2_ Adsorption Applications

The specific surface areas of the TpPa-1 samples synthesized via batch and flow methods were investigated using N_2_ adsorption–desorption isotherms at 77 K (Figure S1). All samples exhibited type I isotherms characteristic of microporous materials, with a sharp uptake at low relative pressures (P/P_0_ < 0.1). The specific surface areas were determined using the BET method, and pore size distributions (Figure S2) were derived from the adsorption branch using nonlocal density functional theory (NLDFT) assuming a slit-pore model. The results are summarized in Table 3.

**Table 3:** Textural Properties of TpPa-1 COFs Synthesized and Evaluated in This Work

				CO_2_ quantity adsorbed (cm^3^ g^–1^ STP)	
Solvent	BET SA with standard deviation (m^2^ g^–1^)	Pore volume (cm^3^ g^–1^)	Main pore size (Å)	273 K	298 K
Dioxane	1035 ± 217	0.549	11.8	97	61
Cyrene	94 ± 34	0.236	14.1	24	13
TEC	201 ± 83	0.267	14.1	38	21
DMSO	340 ± 52	0.273	14.1	58	36
PC	782 ± 137	0.418	11.8	82	52
Diacetin	179 ± 28	0.208	14.1	42	24
Diacetin flow	418 ± 2	0.310	14.1	56	36

Among the batch samples synthesized using different solvents, variations in surface area and mean pore size were observed. The materials with the highest BET SA were synthesized in dioxane and PC, displaying a mean pore size of approximately 1.2 nm, whereas all other materials with lower SA have a mean pore size of 1.4 nm. The H–H distance across the pore of TpPa-1 calculated from atomic center to atomic center is approximately 1.8 nm, as can be resolved from previous literature.^10^ The primary pore size in our lower crystallinity samples is approximately 1.4 nm, consistent with data from other literature and with the distance across the pore when taking into account the probe radius.^10,29^ More details are provided in Figure S7. TpPa-1 also has interlayer hydrogen bonding, which is enhanced for more crystalline samples, where crystal growth is more complete and regular in the 2D direction, leading to fewer defects and a constriction of the pore neck, further narrowing the primary pore diameter in highly crystalline samples (dioxane and PC) to 1.2 nm. The primary pore diameter, calculated via different DFT calculation modes (Table S3), shows consistent trends, indicating ordered staggered stacking.

Although both PC and dioxane can form highly crystalline TpPa-1 structures, subtle differences still exist in the interlayer arrangement. The dioxane sample exhibits peak splitting in PXRD, consistent with enhanced interlayer slip.^47^ In contrast, the PC sample displayed a more uniform stacking structure. The increased interlayer slip slightly relaxes the pore arrangement, resulting in a marked increase in adsorption capacity in low-pressure zones..^52,53^ Consequently, despite a lower 1/fwhm, the dioxane sample shows a higher effective pore volume.

Flow-synthesized TpPa-1 showed porosity comparable to that of the batch counterpart, but its BET surface area increased by 134%, yielding 418 m^2^ g^–1^. The significant increase in BET SA can be attributed to the larger pore volume together with the altered nucleation and growth behavior under flow conditions. Rapid and homogeneous mixing in flow leads to the simultaneous formation of many nuclei, producing smaller TpPa-1 crystallites. In addition, the particles grow more isotropically in flow, compared to the spiky, diffusion-limited morphology observed in batch. The resulting smaller and more uniform particles provide greater accessible surface area for gas adsorption, explaining the improved BET SA value. This reduction in crystallite size and the shift toward a more isotropic morphology are further evidenced by the XRD patterns, where the decreased intensity and broadening of the (100) reflection in the flow sample correlate with a decrease in long-range crystalline domain size.

To further assess the functional porosity, CO_2_ adsorption measurements were carried out at 273 and 298 K (Table 3). Among the batch-synthesized materials, those prepared in PC and dioxane exhibited the highest CO_2_ uptakes of 82 cm^3^ g^–1^ and 97 cm^3^ g^–1^ at 273 K, respectively, consistent with their higher BET surface areas and smaller mean pore sizes. As expected, samples with lower BET SA showed diminished CO_2_ uptake. The flow-synthesized TpPa-1 demonstrated an improved CO_2_ adsorption capacity, reaching 56 cm^3^ g^–1^ at 273 K and 36 cm^3^ g^–1^ at 298 K, not only surpassing the diacetin batch-prepared sample but also reaching similar performance to the higher performing batch DMSO sample.

### 3.5 Process Performance: Productivity, Reproducibility, and Energy Efficiency

To evaluate the overall performance of the continuous-flow synthesis relative to conventional batch processing, four key indicators were examined: space-time yield (STY), functional space-time yield (FSTY), energy consumption per gram of TpPa-1 produced, and reproducibility of the TpPa-1 product based on its BET SA.

The space-time yield, defined as the mass of TpPa-1 obtained per reactor volume per unit time (g L^–1^ h^–1^), reflects the intrinsic productivity of each process. Under the optimized conditions (150 °C, 5 barg, diacetin solvent), the flow synthesis achieved an STY of 9.22 g L^–1^ h^–1^, approximately 30-fold higher than that under batch condition. This enhancement results from continuous reagent delivery and efficient heat transfer.

To integrate functional performance of the material with productivity of the reactor used for synthesis, a Functional Space-Time Yield metric was introduced, defined as

$$\mathrm{FSTY}={\mathrm{CO}}_{2}\;\mathrm{uptake}\;\left({\mathrm{cm}}^{3}\;g^{-1}\right)\times \mathrm{STY}\;\left(g\;L^{-1}\;h^{-1}\right)$$ 5

yielding units of cm^3^ h^–1^ L^–1^, which represent the volumetric CO_2_ adsorption capacity generated per reactor volume per unit time.

Using the CO_2_ uptake measured at 298 K, according to Table 4, the flow-synthesized TpPa-1 exhibited an FSTY of 331.2 cm^3^ L^–1^ h^–1^, exceeding the batch counterpart more than 40-fold (Figure 4). Moreover, the flow TpPa-1 also outperforms batch TpPa-1 materials synthesized by using the best-performing solvents: dioxane (18.3 cm^3^ L^–1^ h^–1^) and PC (15.6 cm^3^ L^–1^ h^–1^). This demonstrates that the continuous-flow process simultaneously enhances both production throughput and functional utility of TpPa-1, establishing a more meaningful measure to compare batch and flow processes.

**Table 4:** STY and Energy Results

Solvent	STY (g L^–1^ h^–1^)	FSTY (cm^3^ L^–1^ h^–1^)	Energy consumption (kJ g^–1^)
Dioxane	0.3	18.3	170.5
PC		15.6	
Diacetin		7.2	
**Diacetin flow**	**9.2**	**331.2**	**17.6**

**Figure 4:**
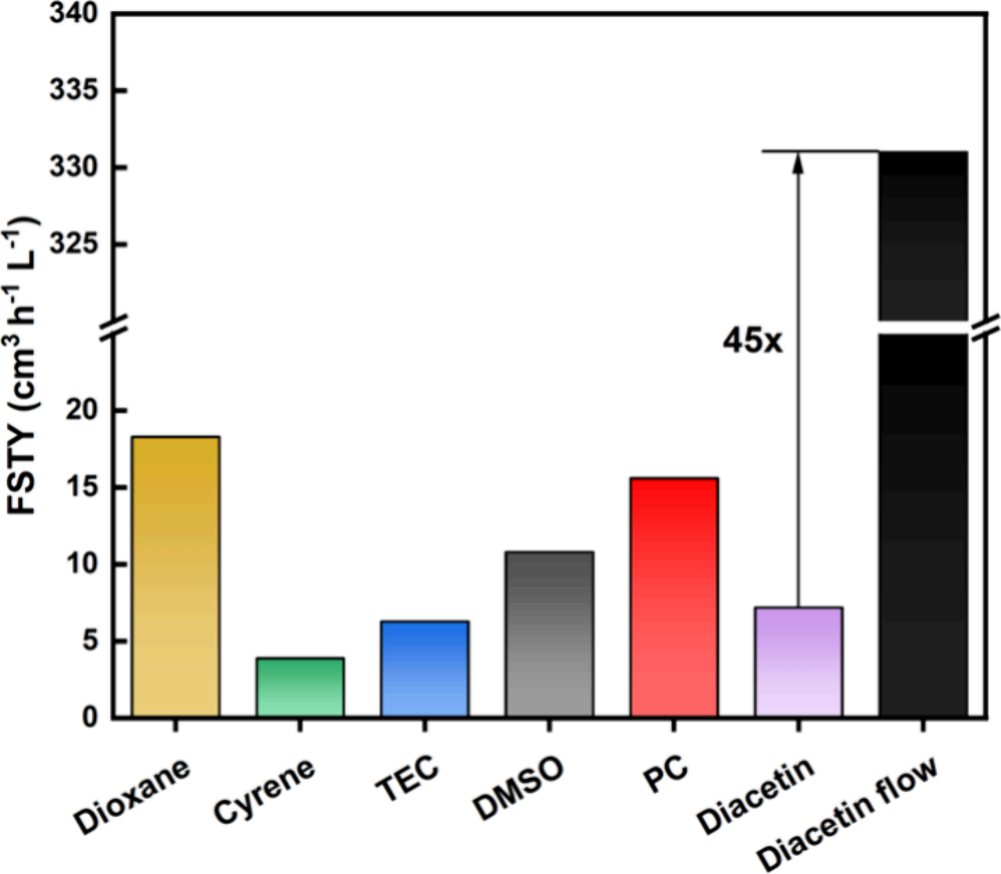
FSTY assessment of powders synthesized by the thermochemical method and flow chemistry.

Energy consumption was estimated by integrating the electrical power input for heating and pumping, normalized to the mass of TpPa-1 produced. At steady state, the flow process required approximately 17.6 kJ g^–1^, representing an energy savings of 89% relative to that of the batch process.

Reproducibility was assessed by conducting three independent syntheses under identical conditions for both batch and flow systems. The flow-derived samples displayed a BET surface area of 418 m^2^ g^–1^, with a standard deviation of <1%, compared with the average standard deviation of approximately 24% across all batch samples. The narrow distribution of BET values confirms excellent reproducibility of the flow method, attributed to the precise control of temperature, residence time, and mixing within the reactor. Collectively, these metrics highlight the advantages of the continuous-flow synthesis of TpPa-1: (i) significantly increased productivity per reactor volume, (ii) high reproducibility and consistent quality, and (iii) lower energy demand.

## 4 Conclusions

This work establishes a practical solvent-selection pathway for sustainable TpPa-1 synthesis by integrating green chemistry principles, Hansen solubility analysis, and free energy solvation modeling. By correlating *ΔΔG*_*solv*_ with crystallization behavior, we identify a moderate solvation window that favors ordered framework growth. Among five less hazardous solvents, propylene carbonate yielded the highest crystallinity in batch synthesis. On the other hand, diacetin provided the best balance of environmental performance and monomer solvation, making it the preferred medium for continuous-flow synthesis.

Transition to a continuous-flow process enabled stable, clog-free operation and produced TpPa-1 with high crystallinity and improved functional performance. Flow operation increased the BET surface area relative to the batch analogue, enhanced CO_2_ uptake, and significantly intensified production. The flow process delivered a 30-fold increase in space-time yield, a more than 40-fold increase in functional space-time yield, excellent reproducibility (<1% standard deviation in BET SA), and an 89% reduction in energy requirement per gram of product. Together, these results demonstrate that solvent design combined with a continuous-flow process provides an efficient, scalable, and environmentally benign route to high-quality COFs and establishes a universal strategy for the production of future sustainable porous materials.

## Supplementary Material



## Notes

Material sources; test information; SMD inputs; PXRD results analysis; flow synthesis attempts; BET results; SEM results; COF powder characterization; space-time-yield (STY) description; functional space time yield (FSTY) description; energy demand (PDF)

## Abbreviations

COFs: Covalent organic frameworks
HSPs: Hansen solubility parameters
*ΔΔG*_*solv*_: Free energy of solvation
SMD: Solvation Model Density
Tp: 2,4,6-trihydroxybenzene-1,3,5-tricarbaldehyde
Dioxane: 1,4-dioxane
PC: propylene carbonate
Pa-1: *p*-phenylenediamine
TEC: triethyl citrate
DMSO: dimethyl sulfoxide
FTIR: Fourier transform infrared
PXRD: powder X-ray diffraction
PFA: perfluoroalkoxy alkane
BET: Brunauer–Emmett–Teller
SA: surface area.
