# Supplementary material for: Structural Modulation of TpPa‑1 Covalent Organic Framework in Flow Synthesis Guided by Green Chemistry Principles
Source: ACS Eng Au. 2026 Apr 17;6(3):427–36. doi: 10.1021/acsengineeringau.5c00114 (PMC13281381; doi:10.1021/acsengineeringau.5c00114)
Supplement: Supplementary file 1 [file eg5c00114_si_001.pdf]

Supporting Information

Structural Modulation of TpPa-1 Covalent Organic Framework in Flow Synthesis Guided by Green Chemistry Principles

Yizhuo Xu,[1#] Nikita Rog,[1#] Catherine Mollart,[2] Ganna Gryn'ova,[2] Abbie Trewin,[3] Cher Hon Lau*[1]

1. School of Engineering, The University of Edinburgh, Edinburgh, EH9 3FB, the United Kingdom of Great Britain and Northern Ireland

2. School of Chemistry, University of Birmingham, Birmingham, B15 2TT, the United Kingdom of Great Britain and Northern Ireland

3. Department of Chemistry, Lancaster University, Lancaster, LA1 4YB, the United Kingdom of Great Britain and Northern Ireland

# These authors contributed equally.

E-mail: cherhon.lau@ed.ac.uk

Contents

1.1 Material sources S2

1.2 Test information S2

1.3 SMD inputs S2

1.4 PXRD results analysis S3

1.5 Flow synthesis attempts S4

2.1 BET results S6

2.2 SEM results S9

2.3 COF powder characterization S10

3.1 Space-Time-Yield (STY) S12

3.1 Functional Space Time Yield (FSTY) S12

3.1 Energy demand S12

1. Additional experimental details

1.1. Material sources

2,4,6-Trihydroxybenzene, 1,3,5-triformylphloroglucinol, diacetin, propylene carbonate and dimethyl sulfoxide were purchased from Fluorochem Ltd. *p*-Phenylenediamine and triethyl citrate were purchased from Thermo Scientific Co., Ltd. Cyrene™ was purchased from Sigma-Aldrich Co. Ltd. 1,4-Dioxane was purchased from Fisher Chemical Co., Ltd.

1.2. Test information

Hansen parameters were obtained using Hansen Solubility Parameters in Practice (HSPiP) software. Powder X-ray diffraction (PXRD) patterns were recorded on Malvern Panalytical Empyrean diffractometer for Cu K-α1 radiation (λ = 1.5406 Å), with a scan speed of 2° min^{-1} and a step size of 0.007° in 2θ. Fourier transform infrared spectroscopy (FTIR) was performed in attenuated total reflectance (ATR) mode on a Nicolet™ iS™ 20 FTIR spectrometer (Thermo Scientific™) with a Smart iTX™ diamond accessory to characterise functional groups over a range of 500 - 4000 cm^{-1}. Scanning electron microscopy (SEM) was tested by a Carl Zeiss SIGMA HD VP Field Emission SEM. A 10 nm-thin layer of gold was sputter-coated on to the samples before imaging. Specific surface areas using the Brunauer–Emmett–Teller (BET) method together with pore size distributions were measured with Autosorb iQ-MP-MP-AG physiosorption analyzer. CO_2 adsorption capacity was measured using Micromeritics TriStar II Plus. X-ray photoelectron spectroscopy (XPS) was tested by a ThermoFisher Nexsa G2 surface analysis system.

1.3. SMD inputs

<u>Tp monomer optimised geometry</u>

21

Energy: -500672.8332623

C	1.38144	0.05942	0.01971
C	0.62986	1.26480	0.00430
C	-0.77705	1.18160	0.00635
C	-1.43103	-0.05747	0.00543
C	-0.63996	-1.23053	-0.01441
C	0.76231	-1.19509	0.00846
O	2.70110	0.11167	0.03211
C	1.61408	-2.40676	-0.00474
O	1.21971	-3.54300	0.01857
O	-1.19714	-2.43756	-0.06884
C	-2.89716	-0.15222	0.10246
O	-3.69186	0.72816	-0.08862
O	-1.54822	2.26878	0.03560
C	1.32921	2.52551	-0.04046
O	2.54554	2.64624	-0.02545
H	0.73420	3.45129	-0.10848
H	2.69096	-2.18596	-0.03675
H	-3.28113	-1.14501	0.41723
H	2.97430	1.06358	0.02223
H	-2.15069	-2.38594	-0.17520
H	-1.03099	3.07204	0.13153

<u>Example input file for obtaining the free energy of solvation using the SMD model</u>

%chk=Tp_monomer_SMD_Dioxane.chk

```
#p M062X/cc-pVTZ SCF=Tight IOP(2/17=4) SCRF=(SMD,Solvent=Generic,Read,dovac,self) INT(grid=ultrafine)

Tp monomer SPE in dioxane using SMD, M06-2X/cc-pVTZ

0 1
Element    x-coordinate    y-coordinate    z-coordinate
…
Element    x-coordinate    y-coordinate    z-coordinate

Surface=sas
Eps=2.2099
EpsInf=2.023222
HbondAcidity=0
HbondBasicity=0.64
SurfaceTensionAtInterface=49.73
CarbonAromaticity=0
ElectronegativeHalogenicity=0
RSolv=0
```

Table S1. Parameters used to define the SMD model for each solvent studied. 1,4-Dioxane and DMSO are pre-parametrised solvents within the Minnesota Solvent Descriptor Database. All other solvents were parametrised as part of this study. Key: Dioxane–1,4-dioxane, TEC–triethyl citrate, PC–propylene carbonate, DMSO–dimethyl sulfoxide, Eps–dielectric constant (298 K), EpsInf–square of the refraction index at 293 K, Alpha–Abraham's hydrogen bond acidity, Beta–Abraham's hydrogen bond basicity, Gamma–the macroscopic surface tension at a liquid-air interface at 298 K divided by 1 cal mol^{-1} Ang^{-2}, Phi–fraction of non-hydrogenic solvent atoms that are aromatic carbon atoms, Psi–fraction of non-hydrogenic solvent atoms that are F, Cl or Br.

Solvent	Eps	EpsInf	Alpha	Beta	Gamma	Phi	Psi
1,4-Dioxane	2.2099[a]	2.023222[a]	0.00[a]	0.64[a]	49.73[b]	0.00[a]	0.00[a]
Cyrene	37.500[b]	2.170318[c]	0.00	0.79[f]	65.70[b]	0.00	0.00
TEC	9.5000[b]	2.089470[d]	0.32[f]	0.83[f]	48.86[b]	0.00	0.00
Diacetin	16.000[b]	2.072160[d]	0.32[f]	0.83[f]	54.77[b]	0.00	0.00
PC	64.920[b]	2.013277[e]	0.00[f]	0.64[g]	59.34[b]	0.00	0.00
DMSO	46.826[a]	2.185371[a]	0.00[a]	0.88[a]	68.15[b]	0.00[a]	0.00[a]

Obtained from: a) Minnesota Solvent Descriptor Database, https://comp.chem.umn.edu/solvation/, accessed 03.02.2025, b) experiment, c) the square of the refractive index in Z. S. Baird, P. Uusi-Kyyny, J.-P. Pokki, E. Pedegert and V. Alopaeus, *Int. J. Thermophysics*, 2019, **40**, 102, d) the square of the refractive indices (for the 1,3- isomer in the case of diacetin) in CRC Handbook of Chemistry and Physics, 95th Ed., Haynes, W. M., Ed., CRC Press: Boca Raton, Florida, 2014, e) the square of the refractive index in the CRC Webbook, f) M. H. Abraham and J. A. Platts, *J. Org. Chem.*, 2001, **66**, 3484–3491, g) J. Jover, R. Bosque and J. Sales, *J. Chem. Inf. Comput. Sci.*, 2004, **44**, 1098–1106. Unlabelled values were deduced from the molecular structure.

1.4. PXRD results analysis

Powder X-ray diffraction (PXRD) patterns were first corrected by subtracting an empty-background profile collected under identical conditions. Crystalline reflections were fitted in local windows by nonlinear least squares using pseudo-Voigt peak profiles to obtain peak positions, FWHM, and integrated intensities. The medium-angle amorphous contribution was modeled with one to two broad pseudo-Voigt peaks and refined together with the crystalline peaks.

1.5. Flow attempts

Table S2. summary of flow synthesis attempts

Attempt	Solvent	Dilution of monomers	Dilution of acetic acid	Residence time	T (°C)	Pressure (bar)	BET SA ($m^2 g^{-1}$)	Comments
1	PC	X1	X1	5 min	120	0	-	Clogged immediately
2	PC	X1	X1	1 min	120	0	-	Clogged immediately
3	PC	X5	X5	5 min	120	0	200	Clogged after 5 minutes of cont. operation
4	PC	X4	X1	5 min	150	0	302	Clogged after 5 minutes of cont. operation
5	PC	X4	X2	5 min	150	0	227	Clogged after 5 minutes of cont. operation
6	Diacetin	X4	X1	5 min	150	0	229	
7	PC	X4	X4	30 min	110	0	243	Clogged after 10 minutes of cont. operation
8	PC	X4	X1	30 min	110	0	288	Clogged after 5 minutes of cont. operation
9	Diacetin	X4	X1	30 min	110	0	292	
10	Diacetin	X2	X1	30 min	110	0	172	Clogged after 10 minutes of cont. operation
11	Diacetin	X4	X1	30 min	150	0	113	
12	Diacetin	X4	X2	30 min	150	5	359	Best performing
13	Diacetin	X4	X1	30 min	120	5	265	

14	Diacetin	X4	X2	30 min	150	5	355	Repeat
15	Diacetin	X4	X2	30 min	150	5	358	Repeat
16	Diacetin	X4	X4	30 min	150	5	228	
17	Diacetin	X4	X8	30 min	150	5	242	
18	Diacetin	X4	X4	1 hour	150	5	348	

2. Supporting results

2.1. BET results

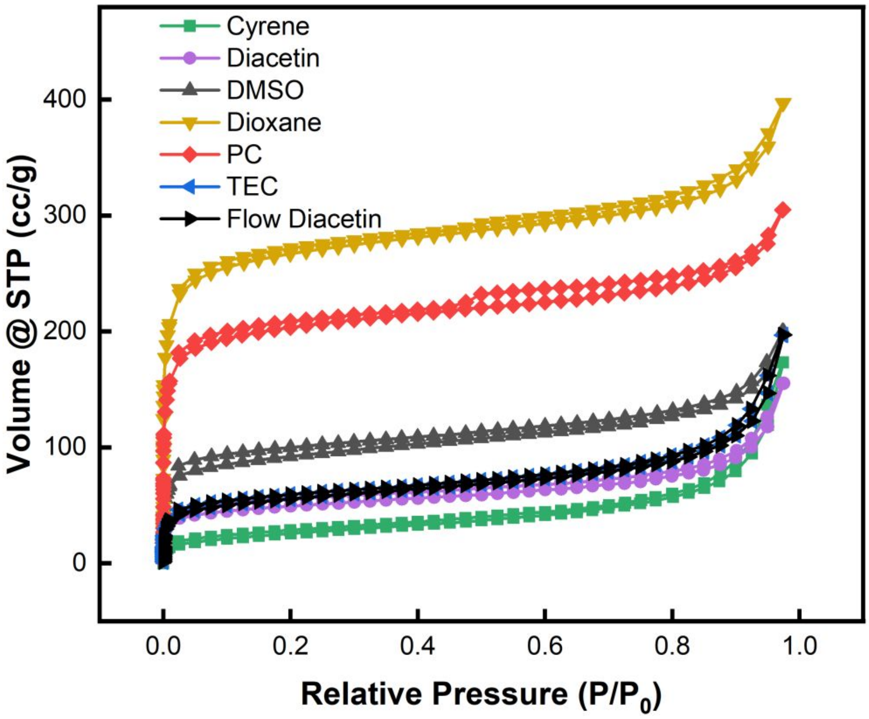

Figure S1. BET N_2 isotherms of TpPa-1 synthesised in the labelled solvents at 77K.

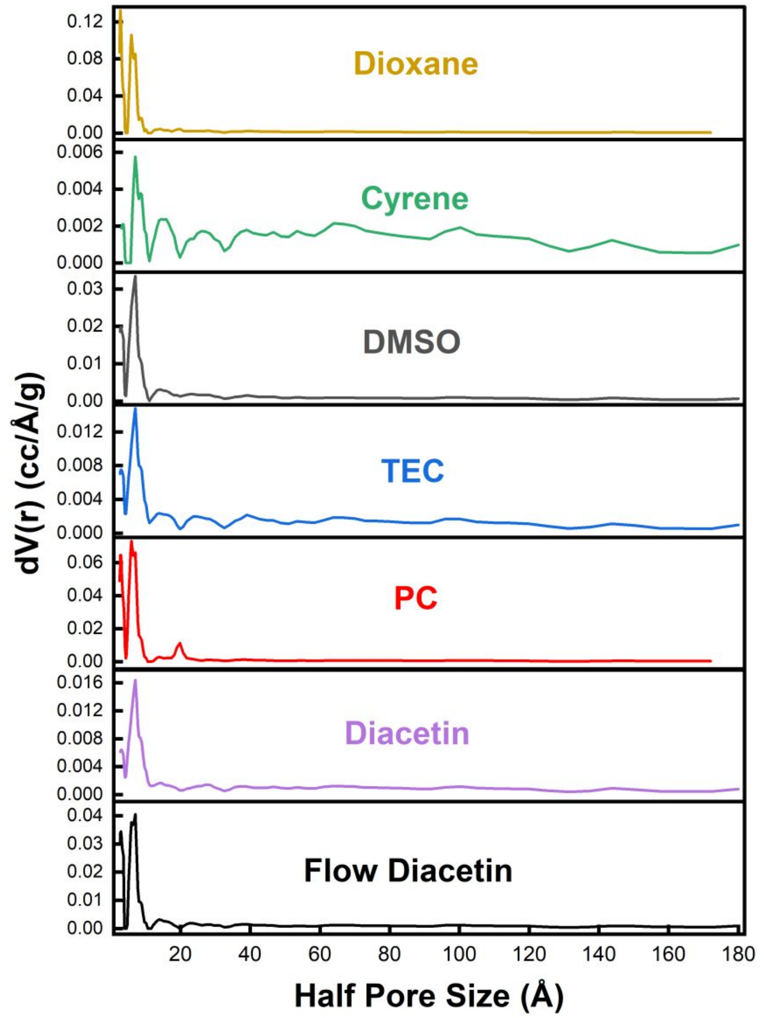

Figure S2. BET pore size distributions of TpPa-1 synthesised in the labelled solvents.

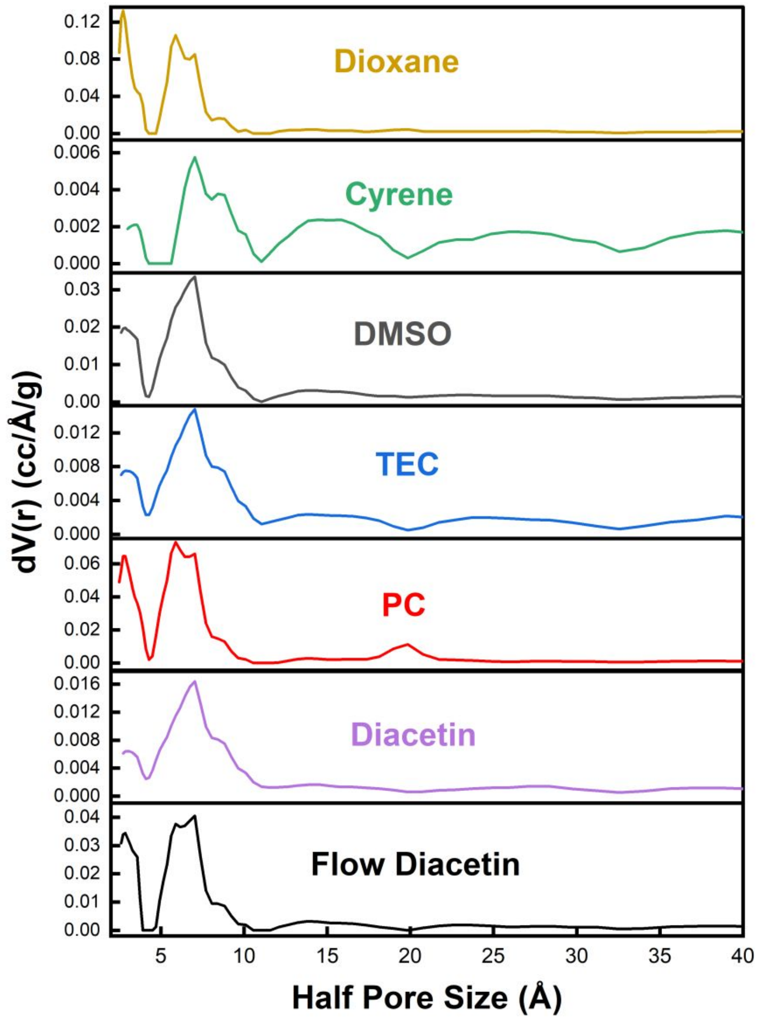

Figure S3. BET pore size distribution of TpPa-1 synthesised in the labelled solvents (zoomed in).

Table S3. Main pore size (Å) using different DFT calculation modes. NLDFT – non-local density functional theory, QSDFT – quenched solid density functional theory.

Solvent	Slit, NLDFT equilibrium	Cylindrical, NLDFT equilibrium	Cylindrical, QSDFT equilibrium
1,4-Dioxane	11.8	14.9	14.5
Cyrene	14.1	-	19.8
TEC	14.1	16.9	17.3
DMSO	14.1	16.9	16.1
PC	11.8	14.9	16.1
Diacetin	14.1	16.9	16.7

2.2. SEM results

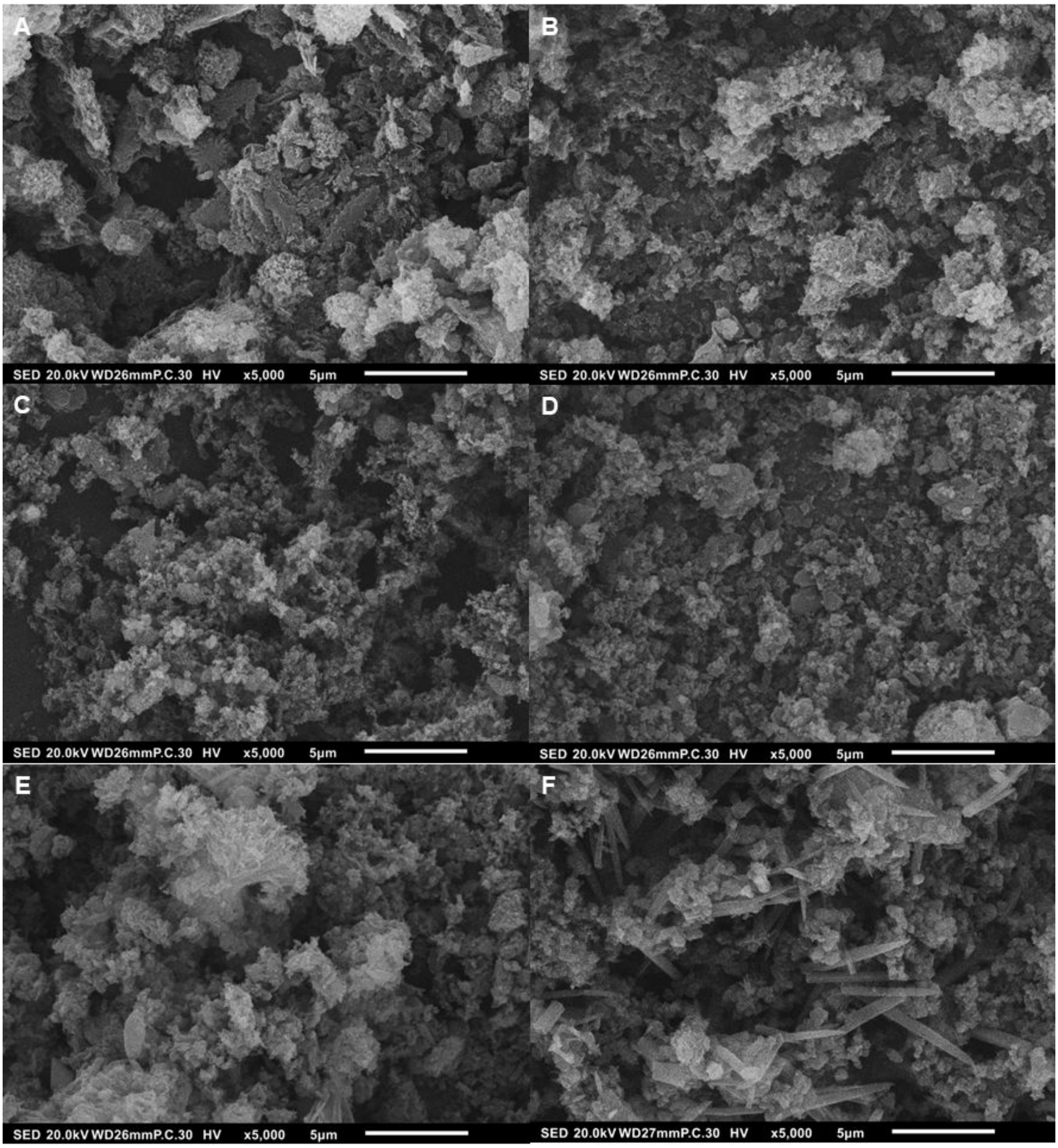

Figure S4. SEM images of TpPa-1 powders synthesized by thermochemical method using (A) Dioxane, (B) cyrene, (C) TEC, (D) DMSO, (E) PC, (F) diacetin.

2.3. COF powder characterization

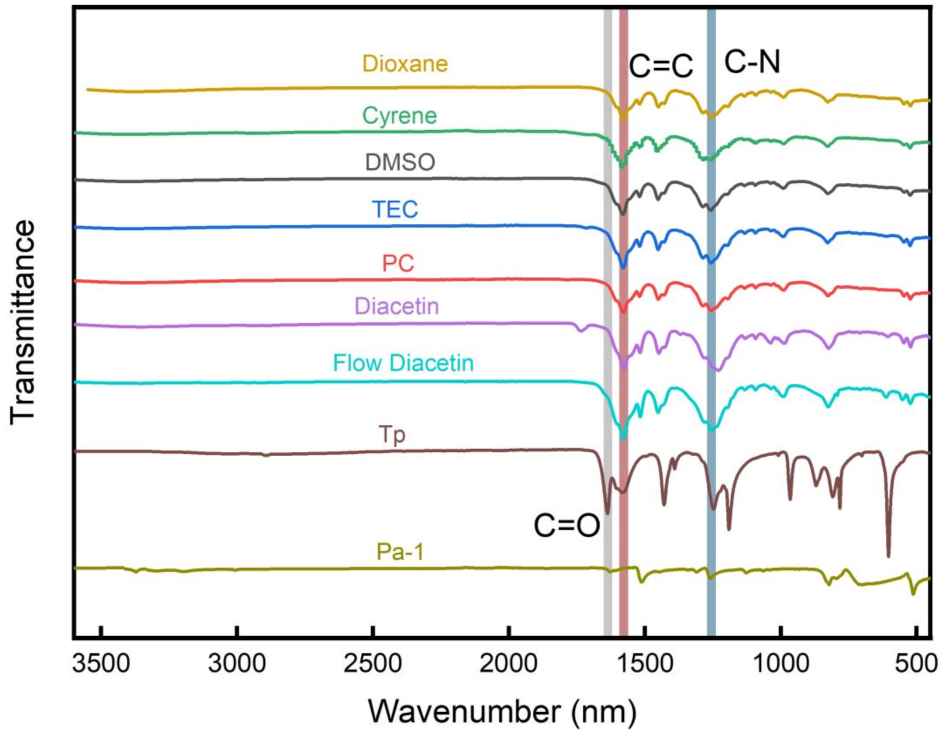

Figure S5. FTIR full scale results of TpPa-1 powders synthesized by thermochemical method in the labelled solvents compared to the Tp and Pa-1 monomers.

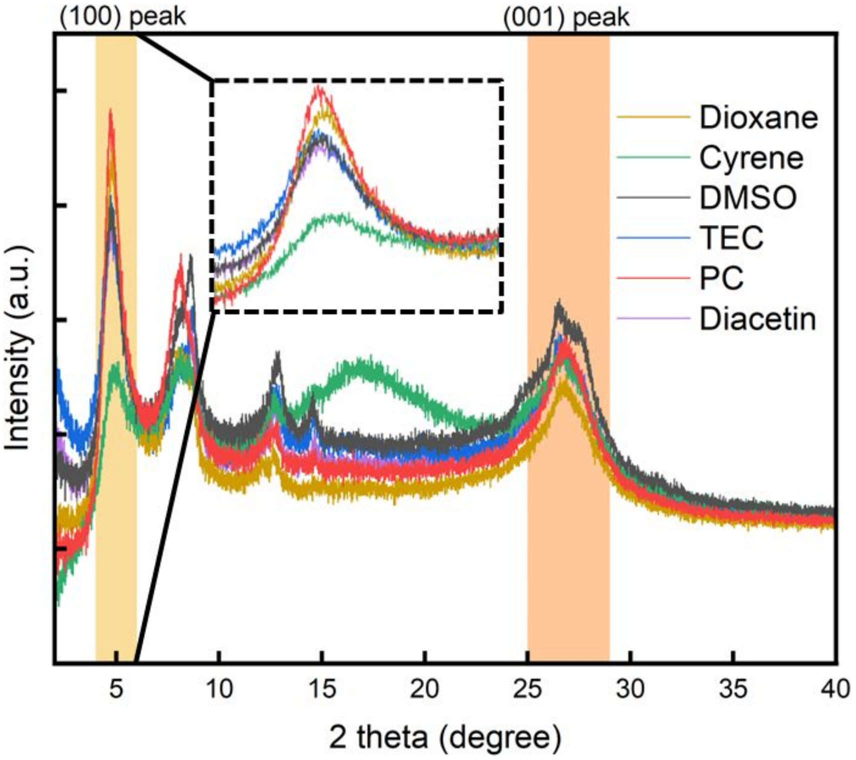

Figure S6. PXRD patterns of TpPa-1 powders synthesized by thermochemical method using the labelled solvents (overlapped + zoomed in). Inset: zoom of the (100) peak.

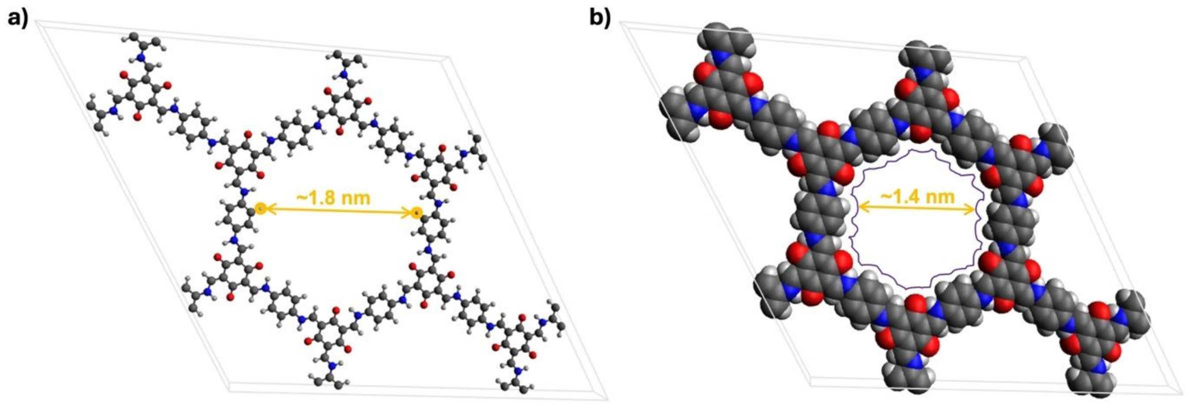

Figure S7. Illustration of the differences between pore diameters obtained from a) theoretical atomic centre to atomic centre measurement and b) an experimental isotherm, where all Van der Waals interactions and the probe radius (indicated by the purple line) are considered.

S3. Sample calculations for STY, FSTY and energy demand

S3.1. STY

Definition:

$$STY = \frac{m_{product}}{V_{reactor} \times t}$$

where $m_{product}$ is the mass of TpPa-1 product, $V_{reactor}$ is the volume of the reactor and t is the reaction time (total reaction time for batch and total time at steady state for flow synthesis).

On average, batch reaction yielded 76 mg of TpPa-1 solid product. The reaction was 3 days long (72 hours). The reactor volume was assumed to be 3.5 mL, which is the sum of volumes of solvent and acetic acid. This volume is the minimum required volume for the batch reactor.

The flow reactor yielded 120 mg of TpPa-1 per 130 minute (2.17 hours) run at steady state. The total volume of the reactor coil was 6 mL.

Sample calculations:

$Batch\ STY = \frac{76\ mg}{3.5\ mL \times 72\ hours} = 0.30\ g\ L^{-1}\ h^{-1}$

$Flow\ STY = \frac{120\ mg}{6\ mL \times 2.17\ hours} = 9.22\ g\ L^{-1}\ h^{-1}$

S3.2. FSTY

Definition:

$$FSTY = CO_2\ uptake \times STY$$

where the CO_2 uptake is the amount of CO_2 adsorbed at 298 K and STY is the space-time yield.

Sample calculations:

For batch diacetin sample: $FSTY = 24\ cm^3\ g^{-1} \times 0.3\ g\ L^{-1}\ h^{-1} = 7.2\ cm^3\ L^{-1}\ h^{-1}$

For flow diacetin sample: $FSTY = 36\ cm^3\ g^{-1} \times 9.22\ g\ L^{-1}\ h^{-1} = 331.2\ cm^3\ L^{-1}\ h^{-1}$

S3.3. Energy demand

Definition:

$$Energy\ demand = \frac{Total\ required\ power \times t}{m_{product}}$$

where $m_{product}$ is the mass of TpPa-1 product and t is the reaction time.

The total required power was considered only at steady-state for the flow reaction. For the batch reaction, we assumed that the power required for freeze-thaw cycles using a vacuum pump is negligible. Additionally, the energy required to heat up an oil bath from room temperature to 120 °C was also not considered to keep it fair with steady-state calculations done for the flow process. Therefore, the following instruments were considered:

- Syringe pump (50 W)
- Coil heater (220 W at 150 °C)
- Hotplate for batch reaction (50 W to maintain 120 °C oil bath)

Sample calculations:

For batch reaction: $Energy\ demand = \frac{50\ W \times 72\ hours}{76\ mg} = 170.5\ J\ g^{-1}$

For flow reaction: $Energy\ demand = \frac{270\ W \times 2.17\ hours}{120\ mg} = 17.6\ J\ g^{-1}$
